# Expression of Potential Cancer Stem Cell Marker ABCG2 is Associated with Malignant Behaviors of Hepatocellular Carcinoma

**DOI:** 10.1155/2013/782581

**Published:** 2013-09-30

**Authors:** Guang Zhang, Zhongxia Wang, Weihuan Luo, Hongbo Jiao, Junhua Wu, Chunping Jiang

**Affiliations:** ^1^Department of Hepatobiliary Surgery, Affiliated Drum Tower Hospital of Nanjing University Medical School, Nanjing, Jiangsu 210008, China; ^2^Department of Hepatobiliary Surgery, Drum Tower Clinical Medical College of Nanjing Medical University, Nanjing, Jiangsu 210008, China; ^3^School of Medicine, Nanjing University, Nanjing, Jiangsu 210093, China

## Abstract

*Background*. Despite improvement in treatment, the prognosis of hepatocellular carcinoma (HCC) remains disastrous. Cancer stem cells (CSCs) may be responsible for cancer malignant behaviors. ATP-binding cassette, subfamily G, member 2 (ABCG2) is widely expressed in both normal and cancer stem cells and may play an important role in cancer malignant behaviors. *Methods*. The expression of ABCG2 in HCC tissues and SMMC-7721 cells was examined, and the relevance of ABCG2 expression with clinical characteristics was analyzed. ABCG2+ and ABCG2− cells were sorted, and the potential of tumorigenicity was determined. Expression level of ABCG2 was manipulated by RNA interference and overexpression. Malignant behaviors including proliferation, drug resistance, migration, and invasion were studied in vitro. *Results*. Expression of ABCG2 was found in a minor group of cells in HCC tissues and cell lines. ABCG2 expression showed tendencies of association with unfavorable prognosis factors. ABCG2 positive cells showed a superior tumorigenicity. Upregulation of ABCG2 enhanced the capacity of proliferation, doxorubicin resistance, migration, and invasion potential, while downregulation of ABCG2 significantly decreased these malignant behaviors. *Conclusion*. Our results indicate that ABCG2 is a potential CSC marker for HCC. Its expression level has a close relationship with tumorigenicity, proliferation, drug resistance, and metastasis ability.

## 1. Introduction

Hepatocellular carcinoma (HCC) is the fifth common cancer in men and the seventh common in women worldwide. Due to its extremely poor prognosis, the deaths and newly diagnosed cases each year are almost equal [1]. Currently, therapeutic strategies for HCC are developing; however, potential curative methods remain surgical resection, transplantation, and radiofrequency ablation [2]. However, according to the widely accepted Barcelona Clinic Liver Cancer (BCLC) staging system, those curative methods are generally limited to early-stage HCC patients, whereas more patients are found with intermediate or advanced stage tumors when diagnosed, thus are not eligible for curative treatment [3]. The effectiveness of noncurative therapies including transcatheter arterial chemoembolization (TACE) and Sorafenib are unsatisfactory, which can only improve overall survival by several months [4]. The dilemma of HCC treatment is largely contributed by the highly malignant behavior of HCC, including early intrahepatic/systemic metastasis and multidrug resistance. 

The theory of cancer stem cells (CSCs) is proposed in recent years. According to CSC hypothesis, the formation and progression of cancers are driven by CSCs which represent a minor population in cancer cells [5]. More importantly, CSCs are considered to be responsible for chemotherapy resistance, metastasis, and postoperative recurrence [6]. Therefore, CSCs may serve as an effective therapeutic target in the treatment of HCC and may improve the current poor prognosis of this disastrous disease. Stem cell phenotypes including self-renewal and the potentiality of differentiation to form heterogeneous cancer cells are deemed to distinguish CSCs from common cancer cells [7]. However, the biomarkers of CSCs are still debatable. Traditional stem cell surface molecules such as CD133, CD44, and CD90 are reported to be markers of CSCs, whereas the results are largely controversial between different studies and tumors from various histological origins [8–10]. On the other hand, side population (SP) cells, which are characterized by efflux of DNA binding dye Hoechst 33342, are also considered as a subpopulation of stem cells in various normal or tumor tissues [11, 12]. Chiba et al. reported that SP cells in HCC xenograft possess extreme tumorigenicity, indicating that this minor population of cells might constitute cancer stem cells in HCC [13]. Further characterization elucidated that ATP-binding cassette (ABC), subfamily G, member 2 (ABCG2), which is widely expressed in various stem cell populations, is highly expressed in SP cells and is responsible for the maintenance of SP phenotype [12]. As an important multidrug resistance transporter, ABCG2 has the capability of efflux various chemotherapy drugs and may contribute to drug resistance of cancer cells [14]. Interestingly, CSCs are also suggested to be responsible for chemoresistance [15]. SP phenotype and chemoresistance strongly imply there is a close association between ABCG2 expression and CSCs maintenance. The conserved expression of ABCG2 in stem cells from both normal tissue and tumor tissues again indicates its important role in stem cell biology [16, 17]. Unfortunately, the effects of ABCG2 expression on CSC-related malignant characteristics are seldom studied.

Our previous studies have reported that the sensitivity to 5-fluorouracil and doxorubicin are negatively correlated with ABCG2 positive rate [18]. In this present study, we investigated the expression of ABCG2 in HCC tissues. Furthermore, we manipulated ABCG2 expression level by RNA interference and plasmid overexpression and studied the effects of ABCG2 expression on HCC malignant behaviors including proliferation, chemoresistance, migration, and invasion.

## 2. Materials and Methods

### 2.1. Patients and Specimens

 Tumor tissues were obtained from 31 patients with pathologically confirmed HCC at Affiliated Drum Tower Hospital of Nanjing University Medical School. All of the patients received curative resection of HCC between April 2009 and April 2011. Demographic and clinical characteristics such as age, gender, HBV infection status, and alpha fetoprotein (AFP) level, were recorded. Clinical stages of HCC were determined by the TNM staging system of the International Union Against Cancer (Edition 6) [19] and BCLC staging system [3]. Tumor differentiation is graded as poor, moderate, or well. Pathological characteristics including tumor number, tumor size, vascular invasion, and positive rate of Ki67 were determined. We also divided patients by Milan criteria as previously described [20]. This study was approved by the Committee of Ethics of Drum Tower Hospital. Written informed consent was obtained from all of the patients.

### 2.2. Immunohistochemistry

Formalin-fixed and paraffin-embedded human samples were firstly cut into 5-*μ*m-thick sections. Then the antigen retrieval was accomplished by deparaffinization, rehydration, and boiling in a microwave oven with citrated buffer. 3% hydrogen peroxide in PBS was used to block the endogenous peroxidase activity and BSA was used to block nonspecific staining. Sections were incubated with rabbit anti-ABCG2 polyclonal antibody (1 : 200, 4477S, Cell Signaling Technology, Danvers, MA) and rabbit anti-Ki67 monoclonal antibody (1 : 900, ab16667, Abcam, Cambridge, MA) at 4°C overnight. The EnVision Kit (DAKO, Carpinteria, CA) was used to detect primary antibody followed by staining with DAB reagent and counterstaining with hematoxylin. At last, the slides were photographed with the microscope (BX50, OLYMPUS, Japan). All slides were evaluated by two independent investigators without knowledge of patients' information. Consensus was reached by discussion if different opinion existed. Placenta tissue was used as positive control, and a section with primary antibody omitted served as negative control.

### 2.3. Cell Culture and Reagents

Human HCC cell line SMMC-7721 was obtained from the Cell Bank of Chinese Academy of Sciences (Shanghai, China). The cells were cultured in Dulbecco's modified Eagle's medium (DMEM) (GIBCO BRL, Gaithersburg, MD) supplemented with 10% fetal bovine serum (FBS) (GIBCO BRL, Gaithersburg, MD) and maintained in 5% CO_2_/95%O_2_ at 37°C. 

Methylthiazolyldiphenyl-tetrazolium bromide (MTT) and doxorubicin were purchased from Sigma Aldrich (St Louis, MO). *β*-Actin antibody (4970S) was obtained from Cell Signaling Technology (Danvers, MA, USA). Horseradish peroxidase (HPR)-conjugated goat anti-rabbit secondary antibody was purchased from MultiSciences Biotech (Hangzhou, China). 

### 2.4. Flow Cytometry

For flow cytometry, cells were detached and incubated with mouse anti-ABCG2 monoclonal antibody (MAB995, IgG2B, R&D Systems, Minneapolis, MN) followed by goat anti-mouse IgG secondary antibody conjugated with FITC (R&D Systems, Minneapolis, MN) for appropriate time. Mouse IgG2B (R&D Systems, Minneapolis, MN) was used as isotype control. A FACSAria flow cytometer (Becton Dickinson, Mountain View, CA) was used to analyze and sort the cells. Cells were sorted by gating FITC-labeled cells compared with isotype control. After sorting, the ABCG2+ and ABCG2− cell fractions were analyzed and purity above 95% was reached.

### 2.5. Tumorigenic Assay

 BALB/c nude mice were maintained in the Animal Experiment Center of Drum Tower Hospital according to the facility's protocol. The protocol of this experiment was reviewed by the local Committee of Ethics. Briefly, mice were randomly divided to receive different number of ABCG2 positive or negative cells subcutaneously (8 × 10^3^, 4 × 10^4^, 2 × 10^5^, 1 × 10^6^, 5 × 10^6^). After inoculation, tumor formation was observed at 1 week, 2 weeks, 4 weeks, and 8 weeks, respectively. The existence and maximum diameter of tumors were measured and recorded.

### 2.6. Small Interfering RNA (siRNA) and Plasmid Transfection

siRNA of ABCG2 was synthesized and provided by RIBOBIO (Guangzhou, China). The construction of pcDNA3.1-ABCG2 expression plasmid was described in our previous paper [21]. For transfection, SMMC-7721 cells were plated in 6-well plate and allowed to grow to 70% confluence. Transfection was done using Lipofectamine 2000 reagent (Invitrogen, Carlsbad, CA) following the manufacturer's guidance.

### 2.7. Western Blotting

Cell lysates were prepared in RIPA lysis buffer (Beyotime, Nantong, China) with a cocktail of protease inhibitors (Roche, Indianapolis, IN). Total protein concentration was determined by BCA reagent following the manufacturer's instruction (Thermo Scientific, Rockford, IL). 20 *μ*g protein was loaded on each lane of the 10% sodium dodecyl sulfate-polyacrylamide gel for electrophoresis. After gel separation, proteins were transferred to 0.45 *μ*m PVDF membranes (Millipore, Bedford, MA). Thereafter, membranes were incubated with primary antibodies overnight at 4°C following blocking membranes with TBS-T containing 5% nonfat milk. Primary antibodies were removed the next day and the membranes were washed with TBS-T. Membranes were then incubated with HRP-conjugated secondary antibodies for 2 hours at room temperature. After washing the membranes, enhanced chemiluminescence (ECL) reagent (Millipore, Bedford, MA) was applied to the membranes. Specific protein bands were visualized by FluorChem FC2 Imaging System (Alpha Innotech, San Leandro, CA).

### 2.8. Reverse Transcriptase Polymerase Chain Reaction (RT-PCR)

Total RNA was reverse transcribed with a commercial cDNA synthesis kit (Takara Biotechnology, Dalian, China) after extracted from cells using TRIzol reagent (Invitrogen, Carlsbad, CA). cDNA templates were amplified with specific primer pairs for ABCG2 and *β*-Actin using ExTaq polymerase and corresponding buffers (Takara Biotechnology, Dalian, China). The primers used are listed as follows: ABCG2 (forward: 5′-TTATCCGTGGTGTGTCTGGA-3′ and reverse: 5′-TTCCTGAGGCCAATAAGGTG-3′), *β*-Actin (forward: 5′-GGCATGGGGTCAGAAGGATT-3′ and reverse: 5′-GAGGCGTACAGGGATAGCAC-3′). Agarose gel electrophoresis was carried out with PCR products to analyze the gene expression.

### 2.9. MTT Assay

The MTT assay was used to determine cell proliferation and doxorubicin sensitivity. Briefly, 5 × 10^3^ cells were planted into 96-well plates. After 24 hours, cells were treated with indicated reagents for different times. 20 *μ*L of MTT (5 mg/mL in PBS) was added and incubated for another 4 hours at 37°C. The MTT formazan precipitate was dissolved in 150 *μ*L of dimethyl sulfoxide after discarding the culture medium. The optical density at 490 nm was measured with a microplate reader (Molecular Devices, Sunnyvale, CA) to estimate the cell proliferation and doxorubicin sensitivity. When testing doxorubicin sensitivity, IC50 value (defined as concentration of drug when cell viability was inhibited by 50%) was calculated.

### 2.10. Wound Healing and Invasion Assay

The wound healing and the transwell invasion assay were performed as previously described  [22]. 1 × 10^5^ cells were incubated for 24 hours after seeded in a 12-well plate followed by serum starvation for more than 12 hours. We disrupted the cell monolayers with a 200 *μ*L pipette tip, and took photographs at 0 and 48 hours in a phase contrast microscope. For the transwell invasion assay, Matrigel (Becton Dickinson, Bedford, MA) diluted with serum-free medium were plated to the upper chamber of transwell inserts (Millipore, Bedford, MA). After the Matrigel clotted, 1 × 10^4^ cells were seeded in the upper chamber in serum-free medium, and the lower chamber was filled with the medium containing 10% FBS as a chemoattractant. After 48 hours, the invaded cells were fixed in methanol and the remaining cells in the upper chamber were scratched with a cotton swab. Invaded cells were stained with crystal violet dye and counted under a microscope. Three random visions were counted to calculate the average number.

### 2.11. Statistical Analysis

 Numeric data were expressed as mean ± SD. Difference between two groups was analyzed by two-tailed Student's *t*-test, and difference among three or more groups was analyzed by one-way analysis of variance multiple comparisons. Categorical data were analyzed by Fisher's exact test. *P* < 0.05 was considered statistically significant.

## 3. Results

**Figure 1:**
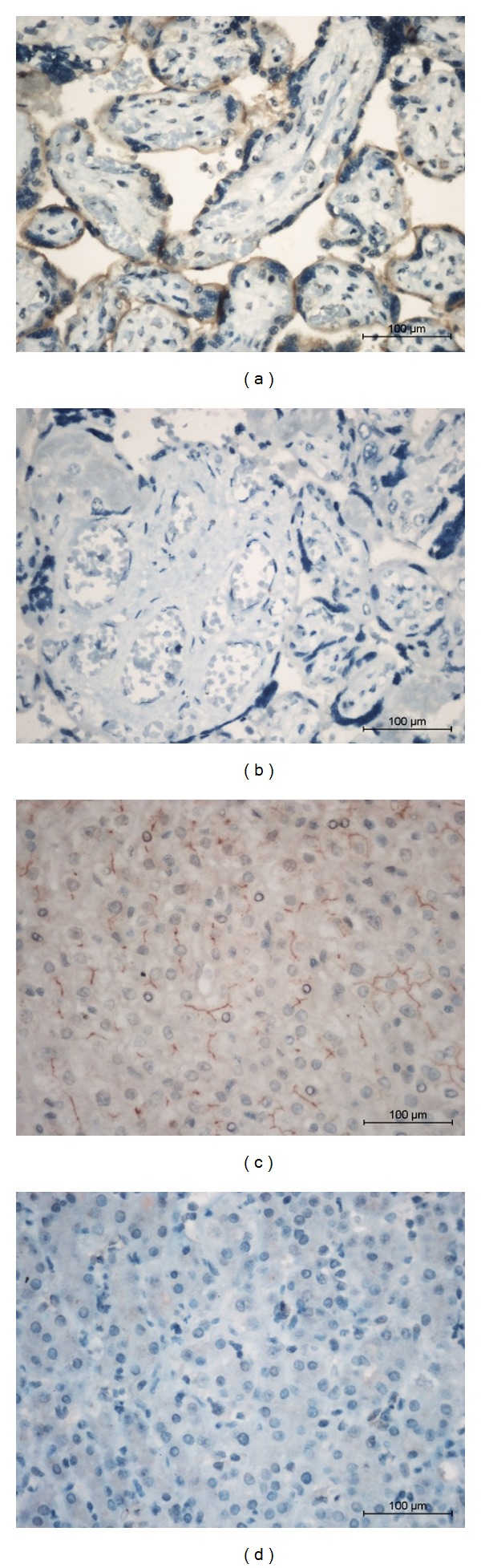
Expression of ABCG2 in human HCC tissues. (a) ABCG2 was expressed in placenta tissues (positive control). (b) Primary antibody omitted placenta tissue slide was set as negative control. (c) Positive ABCG2 expression in HCC tissue. (d) Negative ABCG2 expression in HCC tissue.

*Expression of ABCG2 in HCC Tissue.* Immunohistochemistry was done to determine the positive ratio and expression patterns of ABCG2 in 31 HCC tissues. Placenta tissues were used as positive control. As shown in Figure 1, ABCG2 was mainly expressed on the membrane of villous cytotrophoblast. Negative control showed no positive expression and thus confirmed the specificity of ABCG2 antibody. Twenty one out of the 31 HCC samples were detected with expression of ABCG2. The overall positive ratio was 67.74% (Figures 1(c) and 1(d), 21/31). In ABCG2+ HCC tissue, ABCG2 was expressed on cell membrane, which conformed to the expression pattern of this transmembrane protein (Figure 1(c)). It should be noted that only minor cells (approximately 20%) were stained among the cancer cells.

 When correlated with patients' demographic and clinical information, ABCG2+ group consisted of more male patients compared with ABCG2− group (95.2% versus 60%, *P* = 0.048). Moreover, ABCG2 expression group showed tendencies towards later BCLC stage, more macrovascular invasion, more patients out of Milan criteria, and higher Ki67 index. However, these tendencies did not reach statistical significance (Table 1). 

### 3.1. ABCG2 Was Expressed in a Minor Population in SMMC-7721 Cells

**Figure 2:**
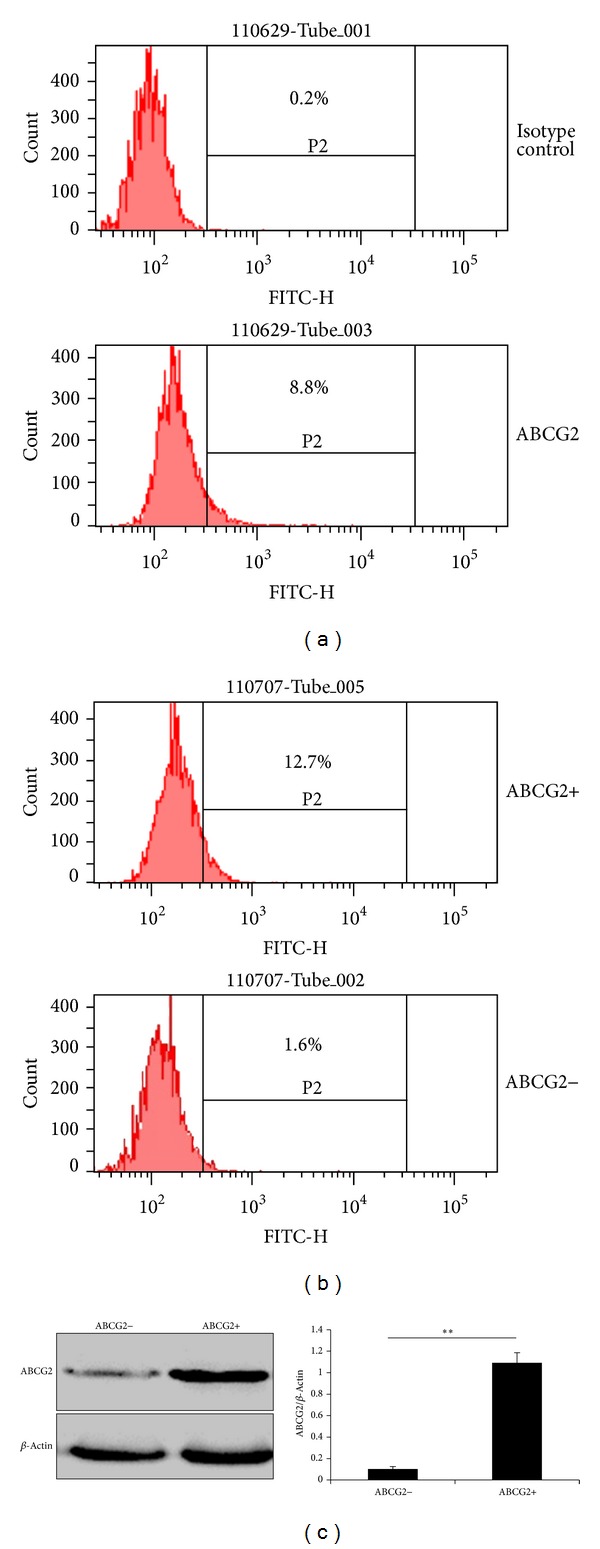
ABCG2 was expressed in a minor population of SMMC-7721 cells. (a) The positive rate of ABCG2 in SMMC-7721 cells determined by flow cytometry was about 8.8% before cell sorting. P2 gate represents ABCG2 positive cells. Mouse IgG2b was used as isotype control. (b) Positive rate of ABCG2 in sorted ABCG2+/ABCG2− cells after 3 passages of culture. (c) Detection of ABCG2 protein level in freshly sorted cells by western blot. *β*-Actin was used as loading control. The optical density of specific bands was quantified and normalized to *β*-Actin. **P < 0.01.

 As shown in flow cytometry assay, the positive ratio of ABCG2 was 8.8% in SMMC-7721 cells (Figure 2(a)). ABCG2+ and ABCG2− cells were sorted by flow cytometer for subsequent experiments. After 3 passages of culture, the positive ratio of ABCG2 in initial positive cells gradually decreased to 12.7%; however, the positive ratio remained low (1.6%) in negative cells (Figure 2(b)). Western blotting confirmed that ABCG2 expression on protein level was significantly higher (about 10 times) in ABCG2+ cells compared with ABCG2− cells.

### 3.2. ABCG2+ HCC Cells Displayed High Tumorigenicity *In Vivo*

In order to evaluate the tumorigenicity of ABCG2+ versus ABCG2− cells, we inoculated subcutaneously 8 × 10^3^, 4 × 10^4^, 2 × 10^5^, 1 × 10^6^, and 5 × 10^6^ ABCG2+ SMMC-7721 cells and the same amount of ABCG2− cells into immunodeficiency mice, respectively. Surprisingly, at 4 weeks after inoculation, all groups of different amounts of ABCG2+ cells formed visible tumors in nude mice while ABCG2− cells failed to establish tumor when inoculated with less than 2 × 10^5^ cells. When only eight thousand cells were grafted into nude mice, ABCG2+ group formed tumors as early as at 2 weeks, and all of the mice developed tumors at 4 weeks after xenograft. However, even inoculated with five times more cells, ABCG2− cells only resulted in one tumor at eight weeks after inoculation (Table 2). Despite the difference of tumorigenicity, tumor sizes were similar between ABCG2+ and ABCG2− groups.

### 3.3. Silencing of ABCG2 Expression Inhibited the Proliferation and Drug Resistance Potential of HCC Cells

**Figure 3:**
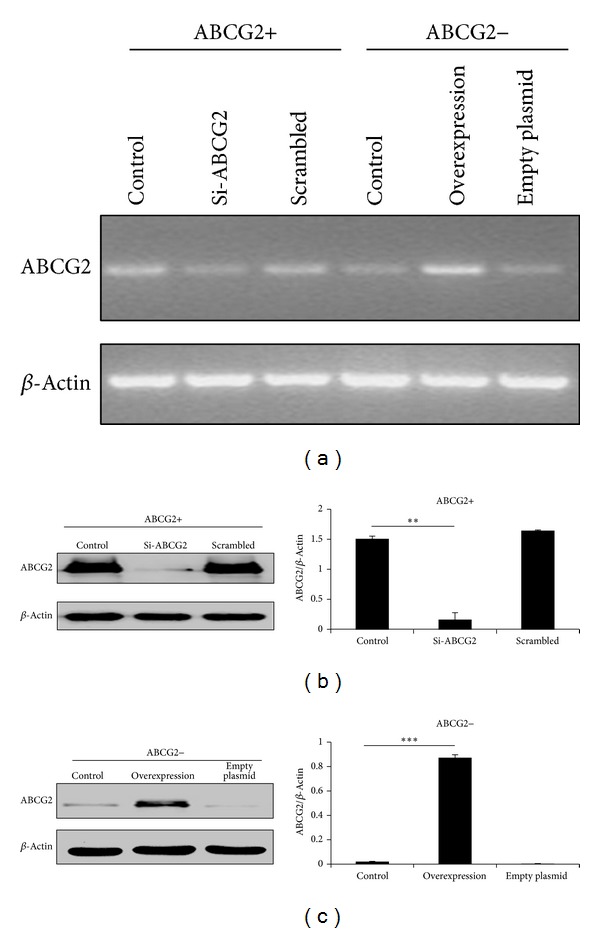
RNA interference and plasmid-mediated overexpression of ABCG2 in SMMC-7721 cells. (a) RT-PCR analysis of ABCG2 mRNA in SMCC-7721 cells after transfection with siRNA or plasmid for 48 h. Normal SMMC-7721 cells were used as blank control. Scrambled siRNA was set as negative control. For overexpression, empty plasmid was transfected as negative control. (b), (c) Western blot analysis confirmed the efficiency of downregulation of ABCG2 by siRNA and upregulation by overexpression, respectively. *β*-Actin served as loading control. The optical density was quantified and normalized to *β*-Actin. **P < 0.01, ***P < 0.001.

**Figure 4:**
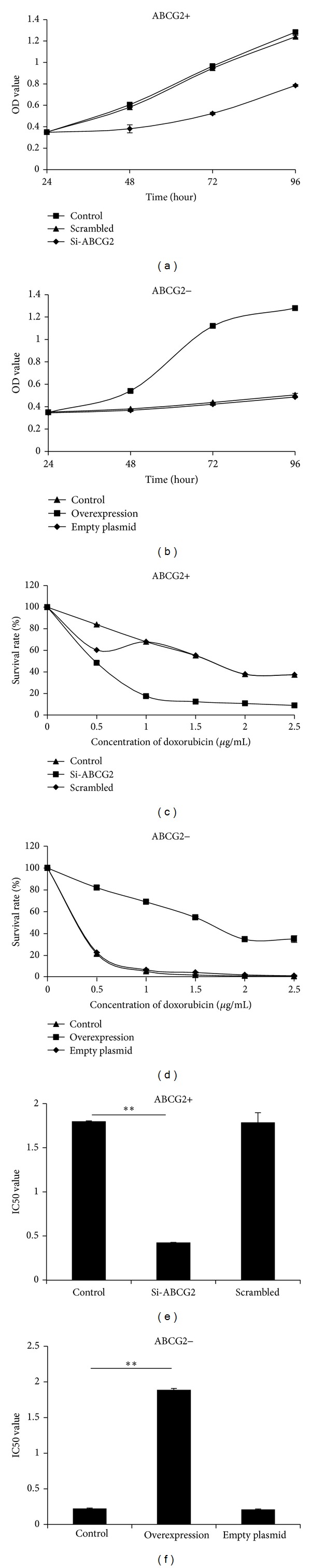
Expression level of ABCG2 correlated with cell proliferation and chemoresistance. (a), (b) Freshly sorted ABCG2+ cells were transfected with ABCG2-specific siRNA. ABCG2− cells were transfected with ABCG2-overexpression plasmid. The proliferation of cells after transfection was compared with untransfected cells and negative control (scrambled siRNA or empty plasmid) using MTT assay after indicated time. (c), (d) The expression level of ABCG2 was manipulated as described above. Survival rates after exposure to indicated dose of doxorubicin for 24 h were determined by MTT assay. (e), (f) IC50 value to doxorubicin was calculated for different groups. **P < 0.01.

As shown in Figure 4, after sorting, ABCG2+ cells exhibited a higher capacity of proliferation and were more resistant to doxorubicin compared with ABCG2− cells. To explore the impact of ABCG2 on proliferation and drug resistance, we used siRNA method to knockdown ABCG2 expression in ABCG2+ HCC separated by flow cytometer. RT-PCR and western blot were employed to verify the efficiency of RNA interference. Transfection of specific siRNA significantly downregulated the expression of ABCG2 at both mRNA level and protein level (Figures 3(a) and 3(b)). Compared with blank control and the scrambled negative control siRNA, the proliferation was significantly inhibited by siRNA-mediated ABCG2 knockdown (Figure 4(a)). Meanwhile, knockdown of ABCG2 enormously sensitized SMMC-7721 cells to cell deaths induced by doxorubicin (Figure 4(c)). The IC50 value decreased from 1.800 *μ*g/mL to 0.426 *μ*g/mL (Figure 4(e)).

### 3.4. Upregulation of ABCG2 Led to Elevated Capacity of Proliferation and Drug Resistance

As the silence of ABCG2 expression decreased the proliferation and drug resistance potential, the effect of upregulation of ABCG2 deserved to be studied. We transfected SMMC-7721 cells with ABCG2-expressing plasmid to transiently upregulate the expression of ABCG2 in ABCG2− cells. After transfection, the change of ABCG2 expression was determined by RT-PCR and western blotting. As shown in Figures 3(a) and 3(c), plasmid transfection significantly increased the expression of ABCG2 at both mRNA and protein levels. In contrast to siRNA, overexpression of ABCG2 significantly enhanced the capacity of proliferation in ABCG2 negative SMMC-7721 cells, which reached similar level with ABCG2+ cells (Figure 4(b)). As expected, transient expression of ABCG2 also desensitized ABCG2− cells to doxorubicin (Figure 4(d)). The IC50 level increased from 0.224 *μ*g/mL to 1.888 *μ*g/mL after transfection of ABCG2 (Figure 4(f)). 

### 3.5. Migration and Invasion Ability of ABCG2+ Cells Was Inhibited by RNAi Knockdown

**Figure 5:**
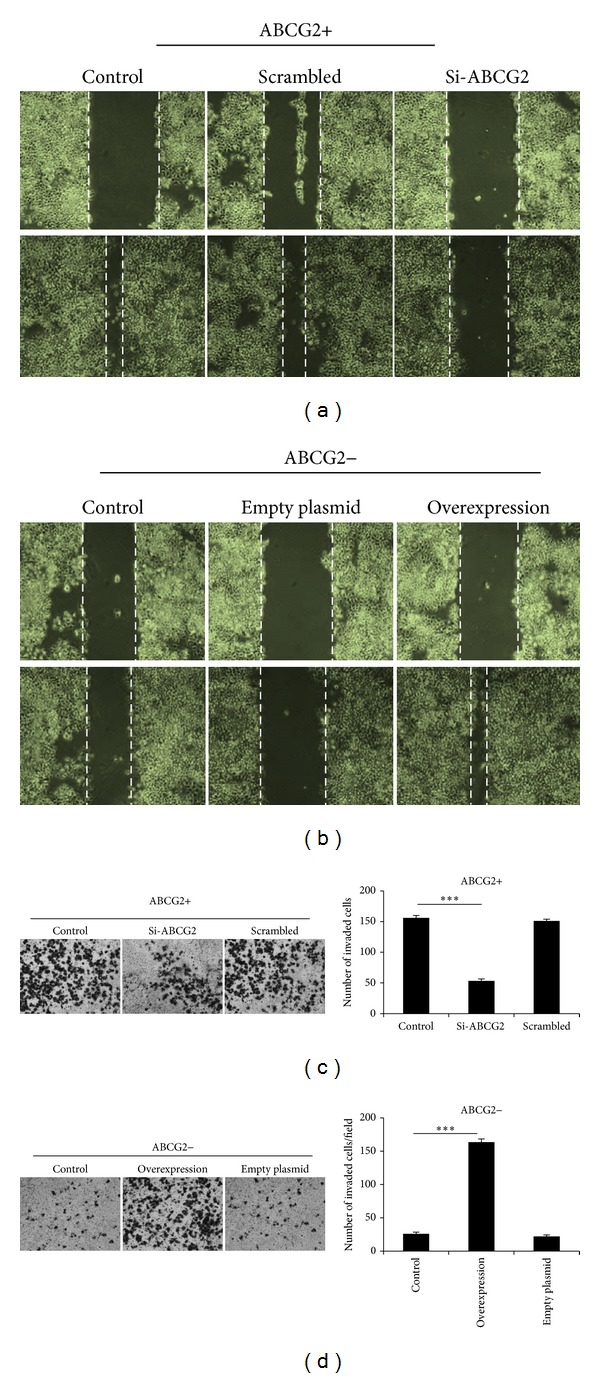
Downregulation or upregulation of ABCG2 had effect on migration and invasion potential in HCC cells. (a), (b) Wound healing assay evaluated the potential of migration of HCC cells. Untreated cells served as normal control. ABCG2+ cells were transfected with ABCG2 siRNA while ABCG2− cells were transfected with ABCG2-expressing plasmid. Scrambled siRNA and empty plasmid were used as negative control. The pictures above represent the wound right after tip scratch. Pictures below showed wound-healing status after 48 h. (c), (d) Results of transwell invasion assay. After 48 h of invasion, cells invaded to the lower chamber were stained and photographed. Invaded cells were counted in three random visions and the average numbers were presented. ***P < 0.001.

In order to investigate the effect of ABCG2 expression on the ability of migration and invasion, we carried out wound healing migration assay and transwell invasion assay. Freshly after sorting, ABCG2+ cells showed higher ability of migration and invasion than ABCG2− cells. However, after transfected with ABCG2-specific siRNA, the migration of ABCG2+ cells was almost totally inhibited in wound healing assay (Figure 5(a)). Similarly, the invasion potential was also tremendously tempered by the downregulation of ABCG2 (Figure 5(c)).

### 3.6. Overexpression of ABCG2 Enhanced Migration and Invasion Capacity of ABCG2− Cells

In contrast to ABCG2+ cells, ABCG2− cells showed low migration and invasion ability. However, after the transfection of ABCG2-expressing plasmid, the scratch gap almost completely healed while the wound in control cells remained obvious (Figure 5(b)). Meanwhile, enhancement of the ability of invasion of ABCG2− cell was also observed in transwell invasion assay (Figure 5(d)). This result indicated that expression of ABCG2 enhanced the ability of migration and invasion of HCC cells.

## 4. Discussion

Despite recent progress in therapeutic strategies, HCC remains incurable to most of patients. Curative resection remains the first choice for the resectable patients  [2]. However, recurrences after surgical treatment occur in about 70% of the patients within 5 years  [23]. Treatment is limited for patients with recurrent disease and the prognosis is poor. For patients with advanced-stage tumors, only noncurative therapies are available and the effect of treatment is unsatisfactory [24]. Lack of effective therapy for HCC is the main reason for the current dilemma of HCC treatment. However, chemotherapy drug resistance and high metastasis potentiality of HCC limited the efficiency of potential adjuvant therapy.

Cancer stem cells have been extensively studied for the last decade. These cells are thought to be along with invasion, metastasis, and drug resistance potentiality [25]. These characteristics could further result in aggressive phenotype of cancer and poor prognosis. Although the CSCs have been studied for years and therapy against CSCs has been innovated, little assistance has been made to clinical practice. Biomarker uncertainty, complex pathogenesis, and nonspecific expression might lead to this result [26]. Just for HCC, biomarkers such as CD133, CD90, and CD44, are all proposed as CSC markers. Recently, ABCG2 is considered as a potential marker of CSCs in HCC since ABCG2 is the maintaining factor of SP cells which have CSC characteristics and highly detrimental behaviors [27]. 

Although CSCs only constitute a small portion of tumor, they may have great influence on the biological behaviors of cancer and may determine the prognosis. It is suggested that the expression of CSC-related markers correlate with poor prognosis of HCC [28]. A newly published meta-analysis focused on the relationship between the CSCs and pathological parameters and found their positive relationships [29]. These results indicate that CSCs may play an important role in the malignant behaviors of HCC and may serve as an effective target for the treatment of HCC. ABCG2 was firstly studied as a stem cell marker in bone marrow [12] and its expression was subsequently detected in various cancers [30–33]. Researchers have also revealed its relevance with high tumor stages and poor prognosis [34–36]. However, this molecular target is relatively less studied in HCC. In agreement with the previous study, we found ABCG2 generally expressed in a small population of HCC cells in both tissue and cell lines. This expression pattern in HCC tissues was in accordance with stem/stem-like phenotype as we found the expression of ABCG2 gradually decreased during passage in positive cells. This phenomenon suggested ABCG2+ cells may possess the ability of differentiation as defined by the characteristics of CSC. Our results supported the notion that ABCG2 may be a potential CSC marker in HCC. Furthermore, positive expression in HCC tissues showed tendencies of association with unfavorable clinical and pathological factors including later BCLC stage, more macrovascular invasion, more patients out of Milan criteria, and higher Ki67 index. Although these tendencies were not statistically significant, which may be at least partly due to the limited sample volume in this study, the prognostic role of ABCG2 deserves further study.

According to CSC hypothesis, CSCs are a minor population of cancer cells that possess the ability to form the huge heterogeneous cancer cell pool. It is reported that CSCs have superior ability of tumorigenicity compared with “normal” cancer cells. Chen et al. separated CD133+EpCAM+ cells in one HCC cell line and found their superior tumor formation capacity [37]. Meanwhile, a recent study found that the SP cells sorted from different HCC cell lines identically possessed strong tumorigenicity. These results reveal the correlation between CSCs and tumorigenicity [38]. In agreement with this theory, we found that ABCG2+ cells sorted by flow cytometry presented significantly higher capability of tumorigenicity than negative cells. As less as eight thousand ABCG2+ cells were able to effectively establish visible tumors in nude mice. This observation supported the relationship between ABCG2 expression and malignant behaviors of HCCs in vivo. However, although ABCG2+ cells are more easy to form tumors, the size of tumors was similar to those that arose from ABCG2− cells. This may be because ABCG2+ tumor-initiating cells gradually differentiated and turned into quiescence during the process of tumor growth. We believe that the dynamic change of ABCG2 during different stages of tumor development is worth studying.

Previous studies elucidated that ABCG2 positive HCC cells have higher capacity of proliferation [39]. However, whether the phenomenon has a direct relationship with the expression level of ABCG2 remains unknown. In this study, we also observed that ABCG2+ cells are more proliferative than negative cells. Moreover, we manipulated the expression of ABCG2 by RNA interference and overexpression. Surprisingly, we found that ABCG2 expression had a direct impact on the proliferation of HCC cells. Upregulation of ABCG2 significantly enhanced proliferation while knockdown of this gene tremendously inhibited the growth of highly proliferative ABCG2 positive cancer cells. Our study also showed that ABCG2 expression in tumor tissue correlated with a tendency of a higher Ki67 index, which is a well-established marker of proliferation. The underlying mechanism may include activation of PI3K/Akt and STAT3 signaling pathways [40, 41]. These results indicated that ABCG2 may directly regulate the proliferation of HCC cells. Downregulating or blocking the activity of ABCG2 may represent an effective method for potential treatment of HCC.

The mechanism why CSCs could escape from chemotherapeutics has been investigated. Researchers found that the CSCs often express high levels of ABC drug transporter and these drug efflux pumps might abolish the accumulation of drugs and then reduce the drug efficiency [27]. As one of the most shining stars of the ABC transporter family, ABCG2 is capable of transporting many substrates including various chemotherapeutics. This characteristic protects tumors from injury of chemotherapy and facilitates drug resistance [27]. In our study, we validated the key role of ABCG2 in chemoresistance. The level of ABCG2 expression correlated with the sensitivity of SMMC-7721 cells to one of the most widely used chemotherapy drugs in HCC-doxorubicin. Manipulation of ABCG2 dramatically changed the effectiveness of this drug on HCC cells. Our results showed that ABCG2 expression level contributed to chemoresistance of HCC. More importantly, ABCG2 may serve as a promising molecular target for the development of adjuvant therapy strategies in combination with traditional chemotherapy.

Among the highly malignant behaviors of HCC, early intrahepatic metastasis and systemic metastasis have largely limited the effect of current treatment of HCC [42]. The potential of metastasis of cancer cells is determined by the ability of migration and invasion of cancer cells [43]. In HCC, invasive characteristics including incomplete tumor capsules, microvascular and macrovascular invasions, and so forth, are correlated with unfavorable prognosis [4]. Strategies targeting migration and invasion are proposed and may improve the prognosis of cancer by reducing metastasis [44]. CSC phenotype of cancer cells is also correlated with the capability of metastasis. It is now accepted that CSCs may be the source of tumor invasion and metastasis [45]. CSC-related biomarkers are also reported to be associated with invasive behavior of cancer in clinical and pathological studies [46, 47]. In this present study, we found that ABCG2+ cells have significantly higher potential of both migration and invasion in vitro. Inhibition of ABCG2 attenuated these malignant behaviors of ABCG2 positive SMMC-7721 cells. Overexpression of ABCG2 endowed low invasive ABCG2− cells with the ability of migration and invasion. These results correlated the level of ABCG2 expression with the potential of metastasis in HCC cells. Again ABCG2 may be an effective target for the prevention of tumor metastasis and corresponding strategies targeting ABCG2 may help improve the metastatic behavior of HCC. 

Our study focused on the role of ABCG2 as a potential CSC marker and its modulatory effect on malignant behaviors of HCC. This present study suggests ABCG2 is expressed in a minor population of HCC cells and ABCG2+ cells manifest some characteristics of CSCs. We also confirm the role of ABCG2 in tumorigenicity, proliferation, drug resistance, migration, and metastasis of HCC. However, further study of ABCG2 in HCC is warranted. Firstly, more pathological specimens should be enrolled to verify the tendencies of association between ABCG2 expression and malignant characteristics of HCC found in this study. Secondly, mechanisms for the changes in biological characteristics should be estimated. At last, particular signaling pathway remains elusive. 

In conclusion, ABCG2 participates in malignant behaviors and may serve as a biomarker of CSCs in HCC. It represents a new therapeutic target for solving the current dilemma of HCC treatment.

## Figures and Tables

**Table 1:** ABCG2 expression and characteristics of patients.

	ABCG2 Expression		*P*-value
	Positive (*n* = 21)	Negative (*n* = 10)	
Age			
>50	14 (66.7%)	7 (70%)	0.992
≤50	7 (33.3%)	3 (30%)	
Gender			
Male	20 (95.2%)	6 (60%)	0.027
Female	1 (4.8%)	4 (40%)	
HBsAg			
Positive	14 (66.7%)	7 (70%)	0.992
Negative	7 (33.3%)	3 (30%)	
AFP (ng/mL)			
≤20	8 (38.1%)	2 (20%)	0.420
>20	13 (61.9%)	8 (80%)	
TNM			
I/II	15 (71.4%)	7 (70%)	1.000
III/IV	6 (28.6%)	3 (30%)	
BCLC			
0/A	10 (47.6%)	7 (70%)	0.280
B/C/D	11 (52.4%)	3 (30%)	
Differentiation			
Poor	3 (16.7%)	1 (10%)	1.000
Moderate-Well	18 (83.3%)	9 (90%)	
Tumor number			
Single	17 (81.0%)	8 (80%)	1.000
Multiple	4 (19.0%)	2 (20%)	
Tumor size			
<5	9 (42.9%)	5 (50%)	1.000
≥5	12 (57.1%)	5 (50%)	
Microvascular invasion			
Present	8 (38.1%)	3 (30%)	0.996
Absent	13 (61.9%)	7 (70%)	
Macrovascular invasion			
Present	6 (28.6%)	1 (10%)	0.379
Absent	15 (71.4%)	9 (90%)	
Milan			
In	8 (38.1%)	5 (50%)	0.700
Out	13 (61.9%)	5 (50%)	
Ki67			
≤20%	9 (42.9%)	6 (60%)	0.458
>20%	12 (57.1%)	4 (40%)	

AFP: *α*-fetoprotein; TNM: TNM staging system of the International Union Against Cancer; BCLC: Barcelona Clinic Liver Cancer staging system; Milan: Milan criteria.

**Table 2:** Difference of tumorigenic ability between ABCG2+ and ABCG2− cells.

Cell phenotype	Cell number	Number of tumors established/total number of mice inoculated (maximum diameter of tumors (cm))			
		1 week	2 weeks	4 weeks	8 weeks
ABCG2+	5 × 10^6^	4/4 (0.4–0.6)	4/4 (0.8–1.2)	3/3 (1.6–2.0)	2/2 (1.7–2.1)
	1 × 10^6^	4/4 (0.3–0.5)	4/4 (0.7–1.0)	4/4 (1.2–1.9)	4/4 (0.7–2.0)
	2 × 10^5^	4/4 (0.1–0.3)	4/4 (0.5–0.9)	4/4 (1.0–1.4)	4/4 (1.2–1.5)
	4 × 10^4^	1/4 (0.2)	4/4 (0.5–0.8)	4/4 (0.9–1.3)	3/3 (1.0–2.2)
	8 × 10^3^	0/4	2/4 (0.2–0.5)	4/4 (0.5–1.0)	4/4 (0.6–1.6)

ABCG2−	5 × 10^6^	4/4 (0.2–0.4)	4/4 (0.8–1.1)	4/4 (1.2–1.9)	2/2 (1.3–1.8)
	1 × 10^6^	4/4 (0.2-0.3)	4/4 (0.7–1.0)	4/4 (1.2–1.8)	3/3 (1.3–2.0)
	2 × 10^5^	0/4	3/4 (0.4–0.6)	4/4 (0.6–1.3)	4/4 (0.6–2.5)
	4 × 10^4^	0/4	0/4	0/4	1/4 (0.8)
	8 × 10^3^	0/4	0/4	0/4	0/4
